# Supplementary material for: A classification model of homelessness using integrated administrative data: Implications for targeting interventions to improve the housing status, health and well-being of a highly vulnerable population
Source: PLoS One. 2020 Aug 20;15(8):e0237905. doi: 10.1371/journal.pone.0237905 (PMC7446866; doi:10.1371/journal.pone.0237905)
Supplement: S1 Fig — (DOCX) [file pone.0237905.s001.docx]

**S1 Figure. Flow Diagram of Sample Selection**

Individuals with homelessness indicator

**N = 41,457**

N = 23,239 identified based on ICD codes from APCD

N = 21,722 (52.4%) identified based on ICD codes from Casemix data

N = 300 identified based on Department of Mental Health data

N = 3,237 identified based on Massachusetts Ambulance Trip Record Information System

N = 6,704 identified based on Prescription Management Program data

N = 13,745 based on multiple indicators

Suspected duplicates/out of state residents excluded from sample

**N = 9,194,710**

Individuals with no indicator of homelessness in any data source

**N = 5,009,182**

Unique individuals in All Payer Claims Database (APCD)

**N = 14,245,349**

Analytic sample:

Unique individuals in APCD also having matching record in at least 1 other Chapter 55 dataset

**N = 5,050,639**
