# Supplementary material for: A classification model of homelessness using integrated administrative data: Implications for targeting interventions to improve the housing status, health and well-being of a highly vulnerable population
Source: PLoS One. 2020 Aug 20;15(8):e0237905. doi: 10.1371/journal.pone.0237905 (PMC7446866; doi:10.1371/journal.pone.0237905)
Supplement: S1 Table — (DOCX) [file pone.0237905.s002.docx]

**S1 Table. Full Results of Binary Logistic Regression Model fit on Development Sample**

| **Variable** | **Source** | **AOR** | **95% CI** |
| --- | --- | --- | --- |
| **Age group (vs. age 45-63)** | **Master Demographic** |  |  |
| 11-17 |  | 0.30 | 0.26-0.36 |
| 18-29 |  | 0.75 | 0.70-0.80 |
| 30-44 |  | 0.83 | 0.78-0.88 |
| 64+ |  | 0.69 | 0.65-0.74 |
| Unknown age |  | 1.79 | 0.86-3.74 |
| **Female (vs. male)** | **Master Demographic** | 0.65 | 0.62-0.68 |
| **Race (vs. unknown race)** |  |  |  |
| White, non-Hispanic/Latino |  | 1.30 | 1.20-1.42 |
| Black, non-Hispanic/Latino |  | 2.46 | 2.16-2.8 |
| Asian, non-Hispanic/Latino |  | 2.21 | 1.97-2.48 |
| Hispanic/Latino |  | 1.89 | 1.70-2.09 |
| American Indian or Other |  | 1.51 | 1.31-1.74 |
| **Mother's Industry/Occupation (vs. unknown or missing)** | **Master Demographic** |  |  |
| Management, Business & Financial Operations |  | 0.84 | 0.62-1.13 |
| Professional |  | 0.97 | 0.79-1.19 |
| Service |  | 0.95 | 0.77-1.18 |
| Sales & Related and Office & Administrative |  | 1.07 | 0.86-1.32 |
| Farming, Forestry, Fishing; Construction & Extraction; Installation, Repair & Maintenance; Production; and Transportation & Material Moving |  | 1.31 | 1.08-1.58 |
| Military |  | 3.84 | 0.40-37.16 |
| Homemakers |  | 1.72 | 1.52-1.96 |
| Other non-paid workers |  | 1.33 | 0.90-1.97 |
| **Identified as Veteran in PDMP** | **PDMP** | 1.59 | 1.18-2.13 |
| **MassHealth member** | **APCD** | 3.25 | 3.08-3.43 |
| **Any claim with ICD9/ICD10 diagnosis code for alcohol abuse** | **APCD** | 1.45 | 1.34-1.57 |
| **Any claim with ICD9/ICD10 diagnosis code for drug abuse** | **APCD** | 1.38 | 1.21-1.58 |
| **Any claim with ICD9/ICD10 diagnosis code for injection drug use** | **APCD** | 1.32 | 1.09-1.60 |
| **Any outpatient, emergency department or hospital discharge record with ICD9/ICD10 code for drug treatment** | **Casemix** | 3.16 | 2.53-3.94 |
| **Any claim for methadone treatment** | **APCD** | 0.84 | 0.69-1.02 |
| **Any claim indicating presence of opioid use disorder** | **APCD** | 0.82 | 0.69-0.98 |
| **Any claim indicating substance abuse treatment** | **APCD** | 1.73 | 1.46-2.05 |
| **Any claim indicating Screening, Brief Intervention and Referral to Treatment** | **APCD** | 0.68 | 0.54-0.86 |
| **Any record of receipt of BSAS ATS/Detox Treatment** | **BSAS** | 1.42 | 1.23-1.64 |
| **Any record of receipt of BSAS Case Management** | **BSAS** | 1.39 | 1.08-1.79 |
| **Any record of receipt of BSAS County Corrections Treatment** | **BSAS** | 1.38 | 1.03-1.87 |
| **Any record of receipt of BSAS Methadone/OBOT** | **BSAS** | 1.09 | 0.89-1.33 |
| **Any record of receipt of BSAS outpatient treatment** | **BSAS** | 0.87 | 0.76-0.99 |
| **Any record of receipt of other BSAS services** | **BSAS** | 1.34 | 0.75-2.41 |
| **Any record of receipt of BSAS post-Detox treatment** | **BSAS** | 1.94 | 1.64-2.3 |
| **Any record of receipt of BSAS Section 35 program services** | **BSAS** | 1.00 | 0.80-1.25 |
| **Any outpatient, emergency department or hospital discharge record with ICD9/ICD10 code for injection drug use** | **Casemix** | 1.23 | 1.03-1.47 |
| **Any outpatient, emergency department or hospital discharge record with ICD9/ICD10 code for opioid use disorder** | **Casemix** | 0.89 | 0.73-1.09 |
| **Any outpatient, emergency department or hospital discharge record with ICD9/ICD10 code for substance abuse** | **Casemix** | 1.95 | 1.76-2.15 |
| **Any claim with ICD9/ICD10 diagnosis code for bipolar disorder** | **APCD** | 1.01 | 1.00-1.01 |
| **Any claim with ICD9/ICD10 diagnosis code for depression** | **APCD** | 1.22 | 1.10-1.34 |
| **Any claim with ICD9/ICD10 diagnosis code for developmental disorder** | **APCD** | 1.00 | 1.00-1.01 |
| **Any claim with ICD9/ICD10 diagnosis code for disassociative disorder** | **APCD** | 1.01 | 1.01-1.02 |
| **Any claim with ICD9/ICD10 diagnosis code for early onset psychiatric disorder** | **APCD** | 1.00 | 1.00-1.00 |
| **Any claim with ICD9/ICD10 diagnosis code for gender identity disorder** | **APCD** | 1.00 | 0.99-1.01 |
| **Any claim with ICD9/ICD10 diagnosis code for impulse control disorder** | **APCD** | 1.01 | 1.00-1.01 |
| **Any claim with ICD9/ICD10 diagnosis code for intellectual disorders** | **APCD** | 1.00 | 0.99-1.00 |
| **Any claim with ICD9/ICD10 diagnosis code for maltreatment** | **APCD** | 1.00 | 1.00-1.00 |
| **Any claim with ICD9/ICD10 diagnosis code for other mental health disorder** | **APCD** | 1.03 | 1.03-1.03 |
| **Any claim with ICD9/ICD10 diagnosis code for mental health related substance use** | **APCD** | 1.00 | 1.00-1.00 |
| **Any claim with ICD9/ICD10 diagnosis code for neuro cognitive disorder** | **APCD** | 1.00 | 1.00-1.00 |
| **Any claim with ICD9/ICD10 diagnosis code for obsessive compulsive disorder** | **APCD** | 0.99 | 0.99-1.00 |
| **Any claim with ICD9/ICD10 diagnosis code for personality disorder A** | **APCD** | 1.01 | 0.99-1.02 |
| **Any claim with ICD9/ICD10 diagnosis code for personality disorder B** | **APCD** | 1.01 | 1.01-1.02 |
| **Any claim with ICD9/ICD10 diagnosis code for personality disorder C** | **APCD** | 1.00 | 0.99-1.02 |
| **Any claim with ICD9/ICD10 diagnosis code for other personality disorder** | **APCD** | 1.01 | 1.00-1.01 |
| **Any claim with ICD9/ICD10 diagnosis code for phobic disorder** | **APCD** | 1.00 | 0.99-1.00 |
| **Any claim with ICD9/ICD10 diagnosis code for psychoses** | **APCD** | 1.36 | 1.26-1.47 |
| **Any claim with ICD9/ICD10 diagnosis code for schizophrenia** | **APCD** | 1.01 | 1.01-1.01 |
| **Any claim with ICD9/ICD10 diagnosis code for self-harm** | **APCD** | 1.52 | 0.94-2.46 |
| **Any claim with ICD9/ICD10 diagnosis code for sleep disorders** | **APCD** | 0.99 | 0.99-1.00 |
| **Any claim with ICD9/ICD10 diagnosis code for somatoform disorders** | **APCD** | 1.00 | 0.99-1.01 |
| **Any claim with ICD9/ICD10 diagnosis code for stress disorders** | **APCD** | 1.01 | 1.01-1.01 |
| **Any claim with ICD9/ICD10 diagnosis code for anxiety** | **APCD** | 0.87 | 0.81-0.92 |
| **Any claim with ICD9/ICD10 diagnosis code for depression** | **APCD** | 0.94 | 0.84-1.05 |
| **Any claim with ICD9/ICD10 diagnosis code for psychosexual disorder** | **APCD** | 1.00 | 0.99-1.02 |
| **Any outpatient, emergency department or hospital discharge record with anxiety diagnosis code** | **Casemix** | 1.07 | 0.98-1.16 |
| **Any outpatient, emergency department or hospital discharge record with ICD9/ICD10 code for bipolar disorder** | **Casemix** | 1.70 | 1.50-1.93 |
| **Any outpatient, emergency department or hospital discharge record with ICD9/ICD10 code for depression** | **Casemix** | 1.73 | 1.60-1.87 |
| **Any outpatient, emergency department or hospital discharge record with ICD9/ICD10 code for developmental disorder** | **Casemix** | 1.38 | 1.09-1.74 |
| **Any outpatient, emergency department or hospital discharge record with ICD9/ICD10 code for disassociative disorder** | **Casemix** | 0.89 | 0.47-1.71 |
| **Any outpatient, emergency department or hospital discharge record with ICD9/ICD10 code for early onset psychiatric disorder** | **Casemix** | 1.11 | 0.95-1.30 |
| **Any outpatient, emergency department or hospital discharge record with ICD9/ICD10 code for gender identity disorder** | **Casemix** | 1.05 | 0.23-4.72 |
| **Any outpatient, emergency department or hospital discharge record with ICD9/ICD10 code for impulse control disorder** | **Casemix** | 2.87 | 1.09-7.57 |
| **Any outpatient, emergency department or hospital discharge record with ICD9/ICD10 code for intellectual disorder** | **Casemix** | 0.50 | 0.34-0.74 |
| **Any outpatient, emergency department or hospital discharge record with ICD9/ICD10 code for maltreatment** | **Casemix** | 2.12 | 1.82-2.47 |
| **Any outpatient, emergency department or hospital discharge record with ICD9/ICD10 code for other mental health disorder** | **Casemix** | 0.95 | 0.84-1.06 |
| **Any outpatient, emergency department or hospital discharge record with ICD9/ICD10 code for mental health related substance abuse** | **Casemix** | 1.89 | 1.74-2.06 |
| **Any outpatient, emergency department or hospital discharge record with ICD9/ICD10 code for neuro-cognitive disorder** | **Casemix** | 1.07 | 0.92-1.24 |
| **Any outpatient, emergency department or hospital discharge record with ICD9/ICD10 code for obsessive compulsive disorder** | **Casemix** | 0.88 | 0.57-1.34 |
| **Any outpatient, emergency department or hospital discharge record with ICD9/ICD10 code for other personality disorder** | **Casemix** | 2.78 | 2.01-3.83 |
| **Any outpatient, emergency department or hospital discharge record with ICD9/ICD10 code for personality disorder B** | **Casemix** | 2.11 | 1.57-2.84 |
| **Any outpatient, emergency department or hospital discharge record with ICD9/ICD10 code for personality disorder C** | **Casemix** | 1.43 | 0.64-3.18 |
| **Any outpatient, emergency department or hospital discharge record with ICD9/ICD10 code for phobic disorder** | **Casemix** | 1.34 | 0.82-2.18 |
| **Any outpatient, emergency department or hospital discharge record with ICD9/ICD10 code for schizophrenia** | **Casemix** | 2.32 | 2.02-2.66 |
| **Any outpatient, emergency department or hospital discharge record with ICD9/ICD10 code for sleep disorder** | **Casemix** | 0.97 | 0.86-1.10 |
| **Any outpatient, emergency department or hospital discharge record with ICD9/ICD10 code for somatoform disorder** | **Casemix** | 4.00 | 0.73-22.06 |
| **Any outpatient, emergency department or hospital discharge record with ICD9/ICD10 code for stress disorder** | **Casemix** | 1.98 | 1.74-2.25 |
| **Any claim with ICD9/ICD10 diagnosis code for medical mental health disorder** | **APCD** | 1.00 | 1.00-1.00 |
| **Any claim with ICD9/ICD10 diagnosis code for medication induced mental health disorder** | **APCD** | 0.99 | 0.97-1.0 |
| **Any record indicating behavioral cancer** | **OCME** | 1.03 | 0.89-1.20 |
| **Any record indicating cancer** | **OCME** | 1.01 | 1.01-1.02 |
| **Any record indicating cancer-related death** | **OCME** | 1.14 | 0.93-1.39 |
| **Any record of emergency department use** | **Casemix** | 0.64 | 0.58-0.70 |
| **Any outpatient, emergency department or hospital discharge record with ICD9/ICD10 code for medical mental health disorder** | **Casemix** | 1.39 | 1.16-1.66 |
| **Any outpatient, emergency department or hospital discharge record with ICD9/ICD10 code for medication induced mental health disorder** | **Casemix** | 0.72 | 0.57-0.92 |
| **Any outpatient, emergency department or hospital discharge record with ICD9/ICD10 code for skin/soft tissue infection** | **Casemix** | 1.65 | 1.52-1.78 |
| **Any DMH record indicating incarceration** | **DMH** | 1.63 | 0.72-3.71 |
| **Any DMH record indicating psychiatric hospitalization** | **DMH** | 0.74 | 0.54-1.03 |
| **Any record of incarceration in DOC state prison** | **DOC** | 1.90 | 1.62-2.23 |
| **Any record of receiving benefits for medical related needs from DVS** | **DVS** | 0.77 | 0.24-2.52 |
| **Any record in APCD substance abuse/mental health records** | **APCD** | 0.45 | 0.37-0.56 |
| **Any record in BSAS** | **BSAS** | 1.50 | 1.32-1.72 |
| **Any record in Casemix mental health records** | **Casemix** | 0.88 | 0.79-0.98 |
| **Any record of service receipt from DMH** | **DMH** | 2.47 | 2.02-3.00 |
| **Any record of service receipt DVS** | **DVS** | 2.49 | 1.89-3.29 |
| **Any record of service in MATRIS** | **MATRIS** | 2.29 | 2.17-2.42 |
| **Any record of prescription in PDMP** | **PDMP** | 0.89 | 0.85-0.95 |

Note: APCD = All Payer Claims Database; BSAS = Bureau of Substance Addiction Services; DMH = Department of Mental Health; DOC = Department of Correction; DVS = Department of Veteran Services; MATRIS = Massachusetts Ambulance Trip Record Information System; OCME = Office of the Chief Medical Examiner; PDMP = Prescription Drug Monitoring Program
