# Supplementary material for: A classification model of homelessness using integrated administrative data: Implications for targeting interventions to improve the housing status, health and well-being of a highly vulnerable population
Source: PLoS One. 2020 Aug 20;15(8):e0237905. doi: 10.1371/journal.pone.0237905 (PMC7446866; doi:10.1371/journal.pone.0237905)
Supplement: Attachment — Submitted filename: PLOSONE_20200122_EM.docx [file pone.0237905.s003.docx]

**Date: 1/22/2020**

**Manuscript #: PONE-D-19-26052**

**Title: Predicting homelessness using integrated administrative data: Implications for**

**targeting interventions to improve the housing status, health and well-being of a highly**

**vulnerable population**

Overall comment: Identifying (classifying or predicting) homelessness is an important topic and has many applications in healthcare and elsewhere. The paper is well-written. I have two major concerns: (1) right type of analysis and (2) focus of the paper. First, in its current format, I think the analysis represents a cross-sectional classification/association type analysis rather than a prediction study. There is no indication whether the variables used occurred before, during, or after experiencing homelessness. As a matter of fact, authors do not have a good indication on when homelessness has occurred. For a prediction or prognosis study, predictors should occur before the event. The timing is important and key in a predictive model. This is not the case in this study. Having said that, I still see value in the study. Classification or identification of risks associated with homelessness is also important. It is up to authors to decide what they want to do and appropriately conduct the analysis. Second, the paper is not focused. I would remove the association between homelessness and other health condition and opioid overdose from the paper. They are irrelevant to the main topic. They can be presented separately and more in depth elsewhere.

The followings are my minor comments:

Abstract:

The main purpose of the study was to develop and validate a model for homelessness prediction. This is an important topic. However, I am not sure why the authors lost the focus and brought into attention association between homelessness and a series of health conditions including opioid overdose. I would suggest the authors discuss these as potential applications of their predictive model and not as the main focus of the manuscript.

Introduction:

Page 3, line 56: Change “service systems” to “publicly-funded systems.”

Page 4, lines 83-86: what is the basis for your assumption? Any citation to validate or explain how you made this assumption and how accurate it could be?

Page 4, lines 86-88: Again, what is the basis for this claim? This is a huge assumption to make. You are basically validating your model not based on actual data on homelessness but based on measures that correlates with homelessness. What are the degrees of correlations? Elaborate. Cite your references for such assumption.

Page 4. Lines 88-91: As I mentioned above, I strongly recommend keeping the paper focus. This paper is about a predictive model of homelessness based on integrated administrative data. Keep the rest for future papers. And stay focus on using various predictive models, make your model parsimonious, validate it properly, show its economic usefulness, etc., etc.

Data and Sample:

Page 5, line 97: What are programmatic decisions?

Page 5, lines 104-105: What are the 15 data sets? What variables is linked with the main Chapter 55 dataset? Why these variables are chosen? Cite your multistage deterministic approach to merge the data across all these datasets (or put it in the appendix).

Page 5, line 114: consider rewording the sentence. So, did I understand this correctly? Among 14,245,349 people included in the Chapter 55 dataset only 5,050,639 had a record in the APCD. Please include a complete and detailed schematic flow diagram of your sample size. This can be included in the appendix.

Measure of Homelessness:

Page 6, line 122-129. Please include number of homeless people identified based on each of the defined criterion in your schematic flow diagram.

Analysis:

Page 10, lines 155: Explain your stratified random sampling. How did you stratify? Did you consider other variables such as age, sex, race/ethnicity to be randomly distributed in both development and validation group?

Page 14, line 233: How did you calculate 14 times? For each correctly identified homeless person, there are 8.5 false positive. Elaborate on this.

Note: I would like to see a table with all related diagnostic measures (i.e. C-statistic, sensitivity, specificity, PPV, NPV, etc.)

Note 2. The specificity of your model is extremely high (95.1%). Could it be because of timing of prediction, meaning that you included variables in your prediction model that was taken after a person experienced homelessness. So, your model actually did not predict homelessness. It assesses the risks of several variables and their associations with homelessness. This is different from prediction. Timing is important in a predictive model. Timing of prediction should be prior to the event. So, in building a predictive model, one should use only predictors that are available prior to being homeless.

Note 3. Please include the following information for the logistic regression model in the appendix:

1. Full name of the abbreviated variables.
2. Diagnostic testing of your regression model.
3. Instead of using “unknown” or “missing” as your reference category, please use more meaningful groups for your categories. For example, for race, use “White” as your reference category.
4. This study covers a wide age-range group (11+). The question about one’s mother’s occupation for certain age group seems irrelevant. And, this variable may change frequently for certain jobs.
5. These data are gathered over time. What if the condition for one person changed? Any thought of including longitudinal (time-variant) variables in your model? In that case probably a generalized estimating equation would be more appropriate than simple logistic model.
6. What do these varibles mean or represent?

**Any record in BSAS**

**Any record in Casemix mental health records**

**Any record in DMH**

**Any record in DVS**

**Any record in Matris**

**Any record in PMP**
